# Discontinuation of adalimumab after attaining disease activity score 28-erythrocyte sedimentation rate remission in patients with rheumatoid arthritis (HONOR study): an observational study

**DOI:** 10.1186/ar4315

**Published:** 2013-09-25

**Authors:** Shintaro Hirata, Kazuyoshi Saito, Satoshi Kubo, Shunsuke Fukuyo, Yasushi Mizuno, Shigeru Iwata, Masao Nawata, Norifumi Sawamukai, Kazuhisa Nakano, Kunihiro Yamaoka, Yoshiya Tanaka

**Affiliations:** 1 The First Department of Internal Medicine, School of Medicine, University of Occupational and Environmental Health, Japan, 1-1 Iseigaoka, Yahatanishi, Kitakyushu 807-8555, Japan

## Abstract

**Introduction:**

Evidences of biologics-free disease control after discontinuing adalimumab (ADA) in rheumatoid arthritis (RA) patients in clinical practice have not been sufficiently investigated. Purpose of this study is to investigate whether disease activity score 28 (DAS28)- erythrocyte sedimentation rate (ESR) remission was preserved after discontinuation of ADA in patients with RA.

**Methods:**

This is an observational but not a randomized controlled study. Among 197 RA patients who initiated with combination of ADA with concomitant MTX, 69 (35%) acquired DAS28 (ESR) < 2.6 for at least 24 weeks. Of those 69 patients, 51 went on ADA discontinuation with their consent, and finally 50 of those with follow-up of > 24 weeks were evaluated. The effect of discontinuing ADA on clinical disease activity, functional disability and radiographic progression were evaluated by DAS28 (ESR), the clinical disease activity index (CDAI) and the simplified disease activity index (SDAI), by a health assessment questionnaire-disability index (HAQ-DI) and by the modified total Sharp score (mTSS), respectively.

**Results:**

The mean age of the participants was 59.5 years with the mean disease duration of 7.1 years. Out of the 50 patients, 29 (58%) were maintained in DAS28 (ESR) < 2.6 at 24 weeks after discontinuing ADA. A logistic regression analysis showed that DAS28 (ESR) at baseline significantly predicted a DAS28 (ESR) < 2.6 maintained after discontinuation of ADA, and a receiver-operating characteristic (ROC) analysis showed that the cut-off value of DAS28 (ESR) at discontinuation was 2.16. The mean HAQ-DI at six months after discontinuing ADA was 0.1 in patients who kept in DAS28 (ESR) < 2.6, and 94.9% (37/39) showed no evidence of radiographic progression (> 0.5 per year of a change in mTSS) at 1 year.

**Conclusions:**

It was possible to maintain DAS28 (ESR) < 2.6 after discontinuation of ADA without functional and radiographic progression and very low DAS28 (ESR) at the discontinuation was associated with successful ADA-free DAS28 (ESR) < 2.6 in patients with RA.

**Trial registration:**

University Hospital Medical Information Network Identifier: UMIN000006669.

## Introduction

The therapeutic strategy against rheumatoid arthritis (RA) has been considerably improved by clinical application of biological agents, including tumor necrosis factor (TNF) inhibitors. In the Treat-to-Target (T2T) statement, Smolen *et al*. reported that remission (REM) or low disease activity (LDA) has become a realistic target to maximize long-term health-related quality of life in patients with RA [1]. T2T recommends that patients who achieve REM or LDA continue medication to maintain these conditions, based on the evidence of non-biological disease-modifying anti-rheumatic drugs (DMARDs). However, corresponding evidence concerning biologics is not sufficiently available to date. Meanwhile, other recommendations announced by the European League Against Rheumatism (EULAR) mention that tapering biological drugs may be considered if a patient is in persistent REM with synthetic DMARDs after tapering glucocorticoids (GCs) [2].

RA is an autoimmune disease that is associated with erosive joint destruction, progressive functional deterioration, systemic complications and high mortality [3], requiring long-term care to avoid long-standing and disabling polyarthritis and persistent deformity, thus leading to substantial socioeconomic burden and impairment of quality of life. In addition, continuous use of biologics not only spurs an increase in the total medical cost but also may increase the risk of serious infections [4].

Furthermore, it may be difficult for patients to accept the idea of never ending treatment. This is one of the biggest obstacles for the acceptance of biologics. Other patients may become skeptical about continuing treatment once they reach clinical remission. To date, rheumatologists have encouraged patients to continue treatment without clear evidence to support this practice.

The HONOR (Humira discontinuation without functional and radiographic damage progressioN follOwing sustained Remission) study was conducted to elucidate clinical, functional and radiographic outcomes after discontinuation of adalimumab (ADA). Fifty patients with either early or established RA participated after their stable disease activity score using 28 joints based on erythrocyte sedimentation rate (DAS28 (ESR)) <2.6 induced with ADA with concomitant methotrexate (MTX) was maintained for at least 24 weeks. The purpose of this study is to investigate whether ADA-free disease control is possible in patients with RA to address three questions: 1) what characterizes patients who can retain DAS28 (ESR) <2.6 even after discontinuation of ADA; 2) what is the effect of ADA discontinuation on radiographic progression as well as functional disability; and 3) whether it is effective to re-administer ADA to patients with exacerbation.

## Methods

### Patients

Male and female adult patients (age >18 years) who met the 1987 American College of Rheumatology (ACR) criteria for RA [5] initiated ADA treatment between July 2008 and April 2011. Treatment with ADA 40 mg bi-weekly was initiated towards DAS28 (ESR) <2.6 induction in those patients who had an insufficient response to MTX (mean 9 mg/week) whenever appropriate in our clinical practice setting. Patients who had been maintained at DAS28 (ESR) <2.6 with MTX and ADA for at least 24 weeks were enrolled in the discontinuation study with their informed consent. DAS28 (ESR) remission in this study was defined as lower than 2.6 without GCs and non-steroidal anti-inflammatory drugs (NSAIDs) or COX-II inhibitors (coxibs), but with MTX therapy at a stable dose. Patients who achieved DAS28 (ESR) <2.6 and maintained that level for at least 24 weeks went on ADA discontinuation and thereafter were subject to analysis after at least another 24 weeks of follow-up. MTX was maintained at a stable dose after discontinuing ADA during the study period, unless the patient was having exacerbation, defined as DAS28 (ESR) >3.2 during the follow-up period. We decided to use a single value rather than change between two values for the exacerbation criteria for our comprehensive handling. Restarting ADA was recommended for patients with exacerbation and was done with their agreement.

The HONOR study [UMIN000006669] was approved by the ethics review board of the University of Occupational and Environmental Health, Japan. The study was conducted in compliance with the Helsinki Declaration.

### Patient disposition

**Figure 1:**
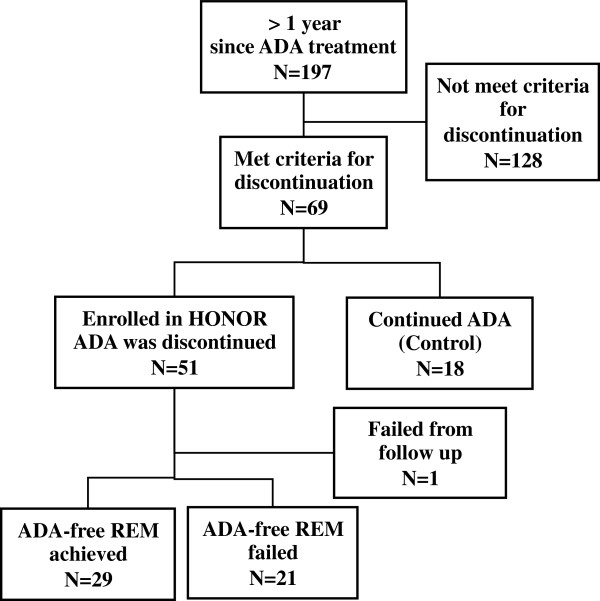
**Flow diagram of patients with rheumatoid arthritis treated with adalimumab and methotrexate, and then discontinued ADA after fulfilling the pre-specified conditions.** ADA, adalimumab; HONOR, Humira discontinuation withOut functional and radiographic damage progressioN follOwing sustained Remission; REM, remission.

Of the 197 patients who initiated ADA (40 mg s.c. every other week) with concomitant MTX (mean ± SD: 8.78 ± 3.02 mg/week) under the conventional Japanese national insurance system between July 2008 and April 2011, 69 patients (35.0%) achieved and maintained a DAS28 (ESR) <2.6 for at least 24 weeks, the criteria for discontinuation (Figure 1). Of those 69 patients, 18 patients who agreed to participate in this study but did not agree to discontinue ADA continued on ADA for at least another 24 weeks (a total of at least one year) in DAS28 (ESR) <2.6 and, thus, served as the control. The remaining 51 patients discontinued ADA, but one patient was excluded from the evaluation because it had not been 24 weeks since she discontinued ADA at the time of data cut-off. All 50 of the eligible patients completed the primary study period of 24 and 52 weeks.

### Efficacy assessments

Clinical assessments were performed at the initiation of ADA treatment, at the withdrawal of ADA, and at 24 and 52 weeks after the withdrawal. The disease activity was assessed by DAS28 (ESR) [6], the simplified disease activity index (SDAI) [7] and the clinical disease activity index (CDAI) [8]. Physical function was assessed at the initiation of ADA treatment, withdrawal of ADA, and 6 and 12 months after withdrawal by using the disability index of the Health Assessment Questionnaire (HAQ-DI) [9]. Radiographs of both the hands and feet were obtained at the initiation of ADA treatment, withdrawal of ADA and 12 months after withdrawal. Radiographs were scored by two independent readers according to the van der Heijde’s modified total Sharp score (mTSS) [10]. For patients who restarted ADA due to exacerbation, efficacy assessments were made at the time of restart.

### Outcome measures

The primary outcome measure was the percentage of patients who maintained DAS28 (ESR) <2.6 at week 24 without ADA. The secondary outcome measures included: the percentage of patients who maintained clinical remission in CDAI (≤2.8) or SDAI (≤3.3); the percentage of patients who maintained clinical low disease activity (LDA) in CDAI (≤10), SDAI (≤11), as well as DAS28 (ESR) (≤3.2) at week 24; the proportions of patients with CDAI, SDAI or DAS28 (ESR) remission at week 52; functional remission as defined by HAQ ≤0.5 at week 24 and/or 52, and with structural remission as defined by the change in mTSS (ΔμTSS) ≤0.5 at week 52 (one year).

### Statistical analysis

Baseline demographic characteristics, disease activity and physical functional status were compared between patients who entered the study and the rest of the patients who initiated treatment with ADA between 24 July 2008 and 4 April 2011 using the Fisher’s exact test or Wilcoxon rank sum test, as appropriate. A similar comparison was made between patients who entered the study and those who did not enter despite DAS28 (ESR) <2.6 with MTX and ADA (the control group).

The associations between variables at the start of ADA discontinuation and 24 weeks maintenance of DAS28 (ESR) <2.6 without ADA were examined in a univariate logistic regression procedure. The variables investigated included duration of ADA administration until the withdrawal, and C-reactive protein (CRP), DAS28 (ESR), SDAI, CDAI and HAQ at the start of ADA discontinuation. A receiver operating characteristics (ROC) curve analysis was conducted to determine cut-off points of variables at the study entry for maintaining DAS28 (ESR) <2.6 for 24 weeks without ADA.

The Wilcoxon rank sum test was employed to test statistically significant differences in disease activity and functional states between sub-groups. The Wilcoxon signed rank test was used to detect statistically significant changes in radiographic progression and functional outcomes over time. All reported *P* values are two-sided and not adjusted for multiple testing. The difference of a *P* value <0.05 was considered to be significant. The last observation carried forward (LOCF) was used for missing clinical or functional values after the start of ADA discontinuation. Liner extrapolation was used to determine ΔmTSS at one year, when patients exacerbated and, thus, restarted ADA or other biological agents. The analyses were performed using JMP^®^ 9.0.3 (SAS Institute Inc., Cary, NC, USA) or Prism^®^ 5.0d (GraphPad Software Inc., San Diego, CA, USA).

## Results

### Baseline characteristics of the patients

Representative baseline characteristics in patients who entered the discontinuation study (n = 51) and those who did not (n = 146) are shown in Table 1. The mean age of the 51 patients was 59.5 years with mean disease duration of 7.1 years, thus indicating that the population included patients with long-established disease. The mean DAS28 (ESR) score was 5.1, implying that most patients had active disease despite treatment with MTX. Furthermore, because the mean annual progression of mTSS between symptom onset and initiating ADA was estimated as 11.5/year, addition of TNF inhibitors to MTX was needed to control joint destruction as well as disease activity. However, mean MTX dose was relatively low (mean ± SD: 8.78 ± 3.02 mg/week), since the MTX dose for RA had been approved as up to 8 mg/week by the Japanese government by February 2011 and thereafter up to 16 mg/week. The study participants had a shorter disease duration, a lower score on HAQ and lower disease activity than the group of patients who did not participate. Patients who met the criteria for discontinuation but did not agree to discontinue ADA served as the control, continuously receiving ADA (n = 18). The study group had a lower score on the HAQ in comparison to the control group (Table 1). Additionally, the positive ratio for rheumatoid factor was 83.7%, for anti-CCP autoantibody was 79.3%, and double negative was 12.7% in all 197 patients. There were no significant differences in autoantibody status among the groups (data not shown).

**Table 1:** **Baseline demographic and disease characteristics of the patients**^**a**^

	**Not maintained DAS28 (ESR) <2.6 for 24 weeks (n = 146)**	**Maintained DAS28 (ESR) <2.6 for 24 weeks (n = 69)**			
		**Discontinued ADA (n = 51)**	***P***^**b**^	**Continued ADA (control) (n = 18)**	***P***^**c**^
Age, year	61.1 ± 11.6	59.5 ± 11.2	0.3910	64.8 ± 10.4	0.0513
Gender, F:M	127:19	40:11	0.1742	14:4	1.0000
Disease duration, year	9.4 ± 10.3	7.1 ± 9.9	0.0356	6.7 ± 10.1	0.7375
RF, U/mL	137.2 ± 248.5	109.0 ± 143.0	0.5539	283.5 ± 550.5	0.1400
TJC, 0 to 28	8.9 ± 6.7	8.1 ± 6.6	0.4014	7.6 ± 5.4	0.9727
SJC, 0 to 28	7.5 ± 5.5	6.4 ± 5.0	0.1770	6.7 ± 4.5	0.6214
PGA, 0 to 100 mm	53.4 ± 24.8	39.0 ± 24.2	0.0008	47.8 ± 24.7	0.2185
EGA, 0 to 100 mm	39.6 ± 22.7^d^	31.5 ± 21.3	0.0398	34.7 ± 19.6^g^	0.4636
CRP, mg/dl	2.89 ± 4.07	2.27 ± 3.71	0.1460	1.61 ± 1.58	0.3817
ESR, mm/hour	51.9 ± 32.4	43.2 ± 33.6	0.0574	45.6 ± 25.0	0.4318
DAS28 (ESR)	5.57 ± 1.27	5.06 ± 1.26	0.0183	5.33 ± 1.25	0.3528
MMP-3, ng/ml	313 ± 363	217 ± 339	0.0301	333 ± 233	0.0005
HAQ, 0 to 3	1.37 ± 0.77^e^	0.91 ± 0.67	0.0003	1.27 ± 0.51^h^	0.0295
MTX, mg/w^f^	8.85 ± 3.11	8.96 ± 2.72	0.8937	9.61 ± 1.97	0.3480

^a^Except where indicated otherwise, values are the mean ± SD; ^b^not maintained DAS28 (ESR) <2.6 for 24 weeks versus maintained DAS28 (ESR) <2.6 for 24 weeks/Discontinued ADA; ^c^maintained DAS28 (ESR) <2.6 for 24 weeks/Discontinued ADA versus maintained DAS28 (ESR) <2.6 for 24 weeks/Continued ADA (control); ^d^N = 72; ^e^N = 143; ^f^the mean ± SD were determined including those without concomitant administration of MTX by handling as zero; ^g^N = 10; ^h^N = 16. CPR, C-reactive protein; DAS28, disease activity score; EGA, evaluator’s global assessment of disease activity; ESR, erythrocyte sedimentation rate; HAQ, Health Assessment Questionnaire; MMP-3, matrix metalloproteinase-3; MTX, methotrexate; PGA, patient’s global assessment of disease activity; RF*,* rheumatoid factor; TJC; tender joint count; SJC, swollen joint count.

### Clinical outcome

**Figure 2:**
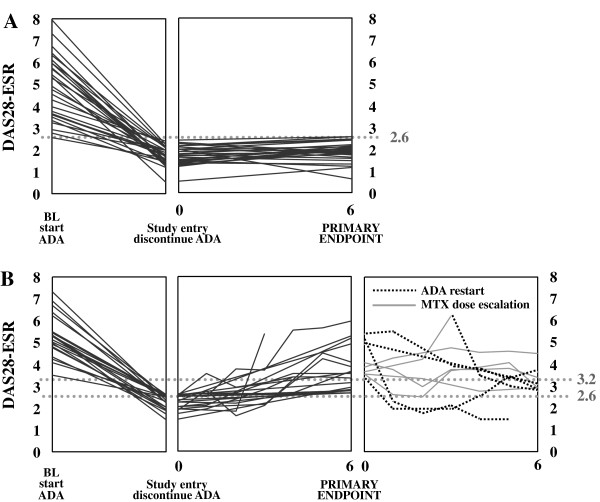
**Time course of DAS28 (ESR) in patients who discontinued adalimumab.** The plots show individual data. **A)** Time course of DAS28 (ESR) at the baseline when adalimumab was initiated, at the HONOR study entry when adalimumab was withdrawn and at the primary endpoint after 24 weeks follow-up in 29 patients who maintained the DAS28 (ESR) <2.6 after discontinuation of adalimumab for at least 24 weeks. **B)** Time course of DAS28 (ESR) at the baseline when adalimumab was initiated, at the HONOR study entry when adalimumab was withdrawn, at the primary endpoint after 24-weeks follow-up and after methotrexate dose escalation and/or adalimumab restart in 21 patients in whom DAS28 (ESR) exceeded 2.6 within 24 weeks of adalimumab discontinuation. DAS28 (ESR), disease activity score by using 28 joints based on erythrocyte sedimentation rate; MTX, methotrexate.

Twenty-nine of the 197 total patients (14.7%) who started ADA, or of the 50 participants (58.0%) who discontinued ADA after maintaining DAS28 <2.6 for at least 24 weeks, achieved a DAS28 (ESR) <2.6 that persisted for more than 24 weeks after the discontinuation (Figures 1 and 2A). However, 21 of the 50 patients (42%) failed to maintain DAS28 (ESR) <2.6 for 24 weeks after ADA discontinuation. Twelve of those patients (24%) experienced exacerbation defined as DAS28 (ESR) >3.2 within a 24-week ADA-free period (Figure 2B). Six of the 12 patients agreed to restart ADA (40 mg/every other week (e.o.w) with a stable MTX dose) while the other six patients did not agreed to restart ADA but three agreed to increase MTX (8 to 14, 10 to 14, and 12 to 14 mg/week, respectively). Among the six patients who restarted ADA, two (33%) attained DAS28 <2.6 again and five (83%) reached DAS28 (ESR) <3.2; however, only one patient (16%) attained DAS28 ≤2.16 at 24 weeks after restarting ADA. Among the three patients who increased MTX but did not restart ADA, none achieved DAS28 <3.2 at 24 weeks after increasing MTX. Among the three patients who did not agreed to restart ADA nor to increase MTX, one achieved DAS28 <3.2 at week 52. Overall, 6/12 patients (50%), who failed to maintain DAS28 (ESR) <3.2 at week 24, in re-induction achieved DAS28 (ESR) <3.2; however, only 2/12 (16.7%) reached DAS28 (ESR) <2.6 and 1/12 (8.3%) reached DAS28 (ESR) ≤2.16 within the following 24 weeks. Restarting ADA due to relapse was not associated with any harmful effects, including skin reaction, serious adverse events, serious infection or withdrawal due to adverse events. On the other hand, patients who restarted ADA had a better response than patients who did not restart ADA but only increased MTX. In the majority of patients, however, the response was not sufficient.

We also focused on ΔDAS28 (ESR) between week 0 and 24 in relation to DAS28 (ESR) <2.6 or <3.2 at week 24 among the 50 participants. When agreement on ΔDAS28 (ESR) <0.6 and DAS28 (ESR) <2.6 at week 24 was assessed, 25 patient fulfilled both, five fulfilled only ΔDAS28 (ESR) <0.6, five fulfilled only DAS28 (ESR) <2.6 and fifteen fulfilled neither. When agreement on ΔDAS28 (ESR) <0.6 and DAS28 (ESR) <3.2 at week 24 was assessed, 30 patients fulfilled both, no one fulfilled only ΔDAS28 (ESR) <0.6, 8 fulfilled only DAS28 (ESR) <2.6, and 12 fulfilled neither. Thus, all patients who did not maintain DAS28 (ESR) <3.2 at week 24 could not maintain ΔDAS28 (ESR) <0.6, as well.

### Identification of predictors for maintaining DAS28 (ESR) <2.6 after ADA discontinuation

A univariate logistic regression analysis was conducted on clinical variables and ADA administration duration before discontinuation in weeks. The CRP, DAS28 (ESR), SDAI and CDAI at study entry were found to affect the maintenance of DAS28 (ESR) <2.6. DAS28 (ESR) gave the best fit with statistical significance (*P* = 0.0004). The logistic regression analysis showed that the ROC analysis indicated that the cut-off point of DAS28 (ESR) at study entry was 2.16. Grouping patients based on this cut-off point revealed that the mean DAS28 (ESR) at 24 weeks was significantly higher for patients in whom disease activity had been DAS28 (ESR) >2.16 at the time of ADA discontinuation (*P* = 0.0017). As a matter of fact, the percentage of patients who maintained DAS28 (ESR) <2.6 at 24 weeks after discontinuation of ADA was 78.1% (25/32) of the patient group with DAS28 (ESR) ≤2.16 at study entry, and only 2.2% (4/18) of the patient group with 2.6 >DAS28 (ESR) >2.16 respectively. The activity of RA with various indices, and each element, such as TJC, SJC, CRP, ESR, PGA and EGA between groups with or without DAS28 (ESR) ≤2.16 at discontinuation is presented in Table 2.

**Table 2:** Time course of composite measures and remission rate stratified by DAS28 (ESR) at discontinuation of ADA

	**DAS28 (ESR) ≤2.16 at discontinuation (n = 32)**				**2.16 <DAS28 (ESR) <2.6 at discontinuation (n = 18)**			
	**Week 0**		**Week 24**		**Week 0**		**Week 24**	
	**mean ± SD**	**median (IQR)**	**mean ± SD**	**median (IQR)**	**mean ± SD**	**median (IQR)**	**mean ± SD**	**median (IQR)**
TJC28	0.06 ± 0.25	0 (0, 0)	1.28 ± 3.27	0 (0, 1)	0.22 ± 0.55	0 (0, 0)	1.39 ± 2.09	0 (0, 2.5)
SJC28	0.03 ± 1.77	0 (0, 0)	0.41 ± 0.98	0 (0, 0)	0.0 ± 0.0	0 (0, 0)	1.17 ± 2.33	0 (0, 1)
PGA (mm)	5.41 ± 6.38	2 (0, 10)	9.75 ± 12.15	4.5 (0.3, 15.8)	8.72 ± 15.08	4 (1.5, 7.5)	16.17 ± 23.63	5 (0.75, 27)
EGA (mm)	2.25 ± 3.62	1 (0, 2.8)	7.22 ± 12.13	1.5 (0, 9.5)	2.50 ± 3.07	2 (0, 3.25)	10.72 ± 16.57	5.5 (0, 10.5)
ESR (mm/hr)	9.78 ± 4.11	9 (6.3, 12)	15.78 ± 10.07	14 (10, 18.8)	24.39 ± 9.05	24.5 (17.5, 32)	39.33 ± 20.55	37 (21.5, 53.75)
CRP (mg/dl)	0.11 ± 0.17	0.04 (0.01, 0.11)	0.35 ± 0.65	0.1 (0.05, 0.30)	0.11 ± 0.22	0.04 (0.03, 0.08)	0.22 ± 0.23	0.15 (0.04, 0.2)
CDAI	0.86 ± 1.00	0.4 (0.03, 1.5)	3.38 ± 5.95	1.0 (0.1, 3.0)	1.34 ± 1.83	0.6 (0.275, 1.7)	5.24 ± 7.32	2.2 (0.075, 9.2)
SDAI	0.97 ± 1.01	0.45 (0.2, 1.8)	3.74 ± 6.23	1.1 (0.3, 4.2)	1.44 ± 1.81	0.8 (0.3, 1.775)	5.45 ± 7.37	2.55 (0.275, 0.2)]
DAS28 (ESR)	1.65 ± 0.34	1.70 (1.45, 2.00)	2.30 ± 1.11	2.00 (1.80, 2.43)	2.41 ± 0.15	2.46 (2.27, 2.54)	3.24 ± 1.14	2.78 (2.54, 3.64)
HAQ	0.14 ± 0.23	0 (0, 0.3)	0.18 ± 0.39	0 (0, 0.1)	0.38 ± 0.38	0.31 (0, 0.66)	0.40 ± 0.44	0.25 (0, 0.78)
mTSS	27.86 ± 61.96	8 (2.5, 23)			64.41 ± 111.33	9 (0.5, 88)		
	n/N	percent	n/N	percent	n/N	percent	n/N	percent
DAS28 (ESR) <2.6	32/32	100%	25/32	78.1%	18/18	100%	4/18	22.2%
DAS28 (ESR) <3.2	32/32	100%	27/32	84.4%	18/18	100%	11/18	61.1%
CDAI ≤2.8	31/32	96.9%	24/32	75.0%	15/18	83.3%	11/18	61.1%
SDAI ≤3.3	31/32	96.9%	23/32	71.9%	15/18	83.3%	11/18	61.1%
Boolean definition	25/32	78.1%	21/32	65.6%	15/18	83.3%	10/18	55.6%

CDAI, clinical disease activity index; CRP, C-reactive protein; DAS28 (ESR), disease activity score by using 28 joints based on erythrocyte sedimentation rate; EGA, evaluator’s global assessment; ESR, erythrocyte sedimentation rate; HAQ, Health Assessment Questionnaire; LOCF, last observation carried forward; mTSS, modified total Sharp score, Boolean definition: ACR/EULAR 2010 definition criteria of clinical remission (TJC ≤1, SJC ≤1, PGA ≤1 cm, CRP ≤1 mg/dl); PGA, patient’s global assessment; SDAI, simplified disease activity index; SJC, swollen joint count; TJC, tender joint count.

As for matrix metalloproteinase-3 (MMP-3) concentration at initiation and discontinuation of ADA, or at 24 weeks after discontinuation, we identified no significant difference between the two groups according to DAS28 (ESR) ≤2.16, 2.6, or 3.2 at week 24, respectively.

### Radiographic outcome

Radiographic progression was assessed in 39 patients at ADA discontinuation through 12 months thereafter. Twelve months had elapsed since ADA discontinuation in 40 of the 50 evaluable patients, but data were missing in four patients. Although a slight increase in the mean ΔmTSS was observed, 94.9% (37/39) showed no evidence of radiographic progression (ΔmTSS ≤0.5) at one year after the discontinuation. The ΔmTSS >0.5 was observed within the first year of ADA discontinuation in two patients, so that their radiographic progression was determined by linearly extrapolating changes using their radiographic scores at exacerbation (data not shown). One of the two patients was a 57 year-old woman with symptom duration of 73 months, and her mTSS was 31 at withdrawal of ADA, which increased to 34 at exacerbation only 13 weeks after withdrawal. The other patient was a 65 year-old woman with symptom duration of 10 months, and her mTSS was 10 at withdrawal of ADA, which exacerbated to 11 at 38 weeks later, although it was a less than clinically relevant radiographic progression (>3/year).

### Functional outcome

The difference in the mean baseline HAQ-DI at the initiation of ADA therapy was not significant between those who maintained in DAS28 (ESR) <2.6 and those who failed (*P* = 0.2693). The mean functional improvement in HAQ-DI observed at the time of ADA discontinuation was maintained at 24 weeks in the sub-group with ADA-free remission, while a slight non-significant worsening was observed in the sub-group that failed to maintain in DAS28 (ESR) <2.6 without ADA.

## Discussion

The present study demonstrated that 29/197 (14.7%) of all the patients with MTX + ADA achieved DAS28 (ESR) <2.6 that persisted for more than six months after ADA discontinuation; this represents 58% of the patients in whom discontinuation of ADA was attempted (with maintenance of MTX). This group maintained ADA-free DAS28 (ESR) <2.6 without functional impairment or radiographic progression over 24 weeks. Although the sample size is limited, the induction of DAS28 (ESR) <2.6 for at least 24 weeks by treatment with ADA and MTX could lead some patients with long-standing RA to discontinuation of ADA, not only in a randomized controlled study setting but also in daily clinical practice.

However, not all patients with stable DAS28 (ESR) <2.6 with ADA and MTX could be maintained in that state after ADA discontinuation. A small increase in the mean DAS28 (ESR) was observed for patients in whom disease activity was higher than the cut-off point of 2.16 at ADA discontinuation. Furthermore, these patients experienced disease exacerbation within 24 weeks after ADA discontinuation at a higher rate than the subgroup with DAS28 (ESR) ≤2.16. This result warrants close monitoring of disease activity after discontinuation and, more importantly, very low DAS28 (ESR) at study entry appears to be a pre-requisite for the successful biologics-free maintenance of DAS28 (ESR) <2.6, which is analogous with the remission induction by Remicade in RA (RRR) study that was previously reported [11]. Additionally, we determined that MMP-3 concentration was not predictive for maintenance of DAS28 (ESR) <2.6 or 3.2 after discontinuation, while it was significantly different between groups with or without discontinuation.

An evaluation of whether restarting anti-TNF therapy would allow recovery of good clinical outcomes in patients who lost response after switching to MTX monotherapy would be valuable. Brocq *et al*. reported that relapsed patients consistently regained remission status after reintroducing ADA and there were no major adverse events in these patients [12]. The HONOR study also found that most of the exacerbated patients achieved a clinical response following ADA restart without any adverse events. On the other hand, dose escalation of MTX tended to be less effective.

Discontinuation of synthetic DMARD has not been recommended, since RA patients in clinical remission had twice as many flare-ups when synthetic DMARDs were discontinued, causing difficulties in reintroducing remission and a halt in damage, whereas similar sufficient evidence has not been seen for the biological DMARDs. Recently, a double-blind, randomized controlled study was carried out to determine the optimal protocol for treatment initiation with ADA with concomitant MTX in patients with early RA (OPTIMA) [13]. In part of the protocol, the outcomes of withdrawal or continuation of ADA were assessed in patients who achieved a stable LDA target after 26 weeks of initially being assigned to treatment with ADA and MTX. Another trial conducted in Germany (HIT HARD) addressed the question of whether an early induction therapy with a subsequent step down strategy leads to a long-term clinical effect in recent onset RA patients in comparison to initial and continued MTX monotherapy [14]. Those trials revealed that the early induction therapy with ADA and MTX followed by withdrawal of ADA led to loss of the response gained with the initial combination treatment in a subgroup of patients in both studies, but not in all patients. However, it should be emphasized that, among patients with very early RA in the OPTIMA and HIT HARD trials, some could be controlled well even with MTX monotherapy.

The targets of long-term RA management are to suppress inflammation, arrest progressive joint destruction and ultimately retain physical function. It is conceivable to alter the progression of RA by modulating the T-cell responses at a very early stage of RA [15]. The results of the RRR and HONOR trials indicate a possibility of halting the disease process in the course of long-standing RA [11]. Both trials used MTX as an anchor drug and then maintenance therapy was considered to be crucial not only for the maintenance of biologics-free disease control, but also in terms of suppressing the immunogenicity of ADA when combined and retaining radiographic inhibitory effects, even after discontinuation [16].

The HONOR study was an observational study with a rather small sample size and a short period of follow-up; thus, whether DAS28 (ESR) <2.6 for at least 24 weeks in patients with long-term RA is sufficient to achieve stable biologics-free maintenance of DAS28 (ESR) <2.6 may require further assessments. Especially, the effect of the dose of concomitant MTX may be of great interest, since in our study we could use a relatively lower dose of MTX due to the limitation of the dose approved by the Japanese national insurance system. The distinctive feature of this study is that the patients had a relatively long disease history (mean 7.1 years) and were not limited only to early RA patients. Yet, it is of clinical as well as medical-economic significance that the present study demonstrated the possibility of ADA-free maintenance of a good condition, such as clinical remission or other comprehensive good outcomes, in a certain patient population with long-standing RA, even in established RA patients.

Of course, it should be emphasized that possible disease control after discontinuation of biological anti-rheumatic drugs is still shown only in preliminary trials, and it should not be used for patients without sufficient informed consent of the risk of worsened outcome. Although the effectiveness of re-administration for the majority of the patients with exacerbation is encouraging, it is more important to evaluate the patients’ condition regularly after discontinuing a TNF inhibitor. Especially, the requirement to re-administer ADA and/or increase MTX should be carefully and repeatedly assessed; otherwise, discontinuation of biologics even after achieving stable DAS28 (ESR) <2.6 should be discouraged. However, these findings may help comfort patients who have to discontinue biologics for any reason, including financial problems or subsequent complications, such as malignancies or infection.

## Conclusions

It was possible to maintain DAS28 (ESR) <2.6 even after discontinuation of the ADA without functional deterioration and/or radiographic damage progression in a certain group of patients. Very low DAS28 (ESR) at discontinuation was associated with successful maintenance of DAS28 (ESR) <2.6 without ADA. Further investigation in a larger population is required to clarify the long-term possibility of ADA-free maintenance of DAS28 (ESR) <2.6 and the medical or socio-economic benefit derived by clinical remission discontinuation.

## Abbreviations

ACR: American College of Rheumatology; ADA: adalimumab; CDAI: Clinical disease activity index; CRP: C-reactive protein; DAS28: Disease activity score using 28 joints; DMARDs: Disease-modifying anti-rheumatic drugs; ESR: Erythrocyte sedimentation rate; EULAR: European League Against Rheumatism; GCs: Glucocorticoids; HAQ-DI: Disability index of Health assessment questionnaire; HONOR: Humira discontinuation without functional and radiographic damage progressioN follOwing sustained Remission; LDA: Low disease activity; LOCF: Last observation carried forward; MMP-3: Matrix metalloproteinase-3; mTSS: modified total Sharp score; MTX: Methotrexate; NSAIDs: non-steroidal anti-inflammatory drugs; RA: rheumatoid arthritis; REM: Remission; ROC: Receiver operating characteristics; SDAI: Simplified disease activity index; T2T: Treat-to-target; TNF: Tumor necrosis factor.

## Competing interests

YT has received grant/research support from Bristol-Myers Squibb, MSD K.K., Chugai Pharma Co., Ltd., Mitsubishi- Tanabe Pharma Co., Ltd., Astellas Pharma Inc., Abbott Japan Co., Ltd., Eisai Co., Ltd. and Janssen Pharmaceutical K.K., Speakers bureau from Mitsubishi-Tanabe Pharma Co., Ltd., Abbott Japan Co., Ltd., Eisai Co., Ltd., Chugai Pharma Co., Ltd., Janssen Pharma K.K., Santen Pharma Co., Ltd., Pfizer Japan Inc., Astellas Pharma Inc., Daiichi-Sankyo Co., Ltd., GlaxoSmithKline K.K., Astra-Zeneca, Otsuka Pharma Co., Ltd., Actelion Pharma Japan Ltd., Eli Lilly Japan K.K., Nippon Kayaku Co., Ltd., UCB Japan Co., Ltd., Quintiles Transnational Japan Co. Ltd., Ono Pharma Co., Ltd. KY has received consultant fees from Pfizer, Chugai Pharmaceutical Co. Ltd. The other authors declare that they have no competing interests.

## Authors’ contributions

SH, KS and YT contributed to the conception and design of the study. All authors (SH, KS, SK, SF, YM, SI, MN, NS, KN, KY and YT) contributed to the acquisition of clinical data. SH, SK, SF and YM participated in radiographic evaluation. SH and YT performed statistical analysis and interpretation of data. All authors (SH, KS, SK, SF, YM, SI, MN, NS, KN, KY and YT) have been involved in drafting the manuscript or revising it critically for important intellectual content. All authors have read and approved the final manuscript.
